# Circulating Interleukin‐6 Predicts Adverse Outcomes in Asians with Hypertrophic Cardiomyopathy

**DOI:** 10.1002/mco2.70463

**Published:** 2025-12-08

**Authors:** Thu‐Thao Le, Shiqi Lim, Chengxi Yang, Jennifer Ann Bryant, Yiying Han, Soon Kieng Phua, Tar‐Choon Aw, Stuart Alexander Cook, Calvin Woon‐Loong Chin

**Affiliations:** ^1^ National Heart Research Institute Singapore National Heart Centre Singapore Singapore Singapore; ^2^ Department of Laboratory Medicine Changi General Hospital Singapore Singapore

1

Dear Editor,

Hypertrophic cardiomyopathy (HCM) represents a heterogeneous cardiac disorder characterized by a wide spectrum of clinical presentations, ranging from asymptomatic cases to those with sudden cardiac death or progressive heart failure. Among its morphological variants, apical HCM (ApHCM) is notably more prevalent in Asian populations compared with Western cohorts. ApHCM is typically associated with a relatively benign clinical course, with lower rates of ventricular arrhythmias and heart failure when contrasted with nonapical forms (Non‐ApHCM) [1]. However, Non‐ApHCM carries a higher risk of adverse outcomes and often demonstrates more extensive myocardial fibrosis and remodeling.

Traditional risk stratification in HCM largely focuses on structural markers and the use of established biomarkers such as N‐terminal probrain natriuretic peptide (NT‐proBNP) and high‐sensitivity troponin T (hsTnT) [2]. Cardiovascular magnetic resonance (CMR) imaging parameters, such as late gadolinium enhancement (LGE), have further enhanced prognostic capabilities. Nevertheless, growing evidence suggests that inflammatory pathways also contribute significantly to myocardial remodeling and disease progression in HCM [3]. Interleukin‐6 (IL‐6), a proinflammatory cytokine, has been implicated in myocardial fibrosis and hypertrophy across multiple cardiovascular diseases. Despite its biological plausibility, the specific prognostic role of IL‐6 in HCM has remained underexplored. This study aimed to investigate whether IL‐6 levels could serve as an adjunctive tool to traditional morphological assessments, particularly in improving risk stratification among Asian HCM patients.

A total of 255 patients with a clinical diagnosis of HCM were prospectively enrolled from the National Heart Centre Singapore between 2013 and 2023 under the Institutional Biobank program (ClinicalTrials.gov NCT02804269). Diagnosis adhered to the 2020 American College of Cardiology/American Heart Association guidelines for HCM [4]. Detailed clinical evaluations, including genetic testing using a targeted cardiomyopathy panel, were performed. Variants were classified following the American College of Medical Genetics and Genomics and the Association for Molecular Pathology guidelines (File S1, Supporting Information).

Serum levels of IL‐6, NT‐proBNP, hsTnT, and C‐reactive protein (CRP) were measured using standardized electrochemiluminescence immunoassays. For biomarker values below the lower limit of detection, an imputed value equivalent to half the detection threshold was assigned. Elevated IL‐6 was primarily defined using the cohort median. ROC analysis with Youden's index identified an optimal cutoff for predicting major adverse cardiac events (MACE) that was nearly identical to the median; therefore, results are presented using the median, with ROC‐based performance metrics reported for validation. CMR imaging was conducted to assess left ventricular (LV) function, myocardial mass, strain, and fibrosis. Replacement fibrosis was quantified using LGE sequences, while myocardial interstitial fibrosis was assessed through extracellular volume (ECV) fraction measurements. Strain parameters were derived from cine imaging using feature‐tracking techniques (File S1, Supporting Information).

The primary outcome was the occurrence of MACE, defined as a composite of ventricular arrhythmias, heart failure hospitalization, new‐onset atrial fibrillation, ischemic stroke, or all‐cause mortality. Patients were followed for a mean duration of 6.7 ± 2.4 years.

Among the 255 patients included in the final analysis, 71 (27.8%) were diagnosed with ApHCM. Patients with ApHCM were significantly older, predominantly male, and exhibited a greater prevalence of metabolic comorbidities, including hypertension and diabetes mellitus (both *p* < 0.01). ApHCM patients had a lower frequency of pathogenic sarcomeric gene mutations compared with Non‐ApHCM patients (25.4 vs. 49.5%).

Non‐ApHCM patients exhibited significantly greater myocardial fibrosis, demonstrated by higher ECV values, and indexed interstitial volumes on CMR. Additionally, Non‐ApHCM patients showed lower LV ejection fractions and worse strain values compared with ApHCM patients. NT‐proBNP and hsTnT levels correlated well with markers of myocardial fibrosis and LV remodeling. IL‐6 levels were elevated in both ApHCM and Non‐ApHCM cohorts and did not show direct associations with CMR imaging parameters, suggesting that IL‐6 may reflect an independent inflammatory risk pathway. Despite the lack of association with imaging markers, patients with elevated IL‐6 had a significantly higher risk of MACE, indicating IL‐6 is associated with adverse outcomes independent of CMR findings. ROC analysis of IL‐6 for predicting MACE yielded an AUC of 0.65 (95% CI 0.57–0.73). The optimal cutoff based on Youden's index was 2.00 pg/mL, which was nearly identical to the cohort median (1.99 pg/mL). As the two were concordant, the cohort median was retained as the primary cutoff in analyses. Patients with IL‐6 concentrations above the cohort median had a twofold higher risk of experiencing a MACE (hazard ratio 2.43; 95% CI, 1.48–3.98; *p* < 0.001).

When stratified by morphological subtypes and IL‐6 levels, Non‐ApHCM patients with high IL‐6 levels emerged as the highest‐risk group, with an event rate of 8.8 per 100 patient‐years. In contrast, ApHCM patients with low IL‐6 levels had the most favorable prognosis, with an event rate of 0.9 per 100 patient‐years (Figure 1).

**FIGURE 1:**
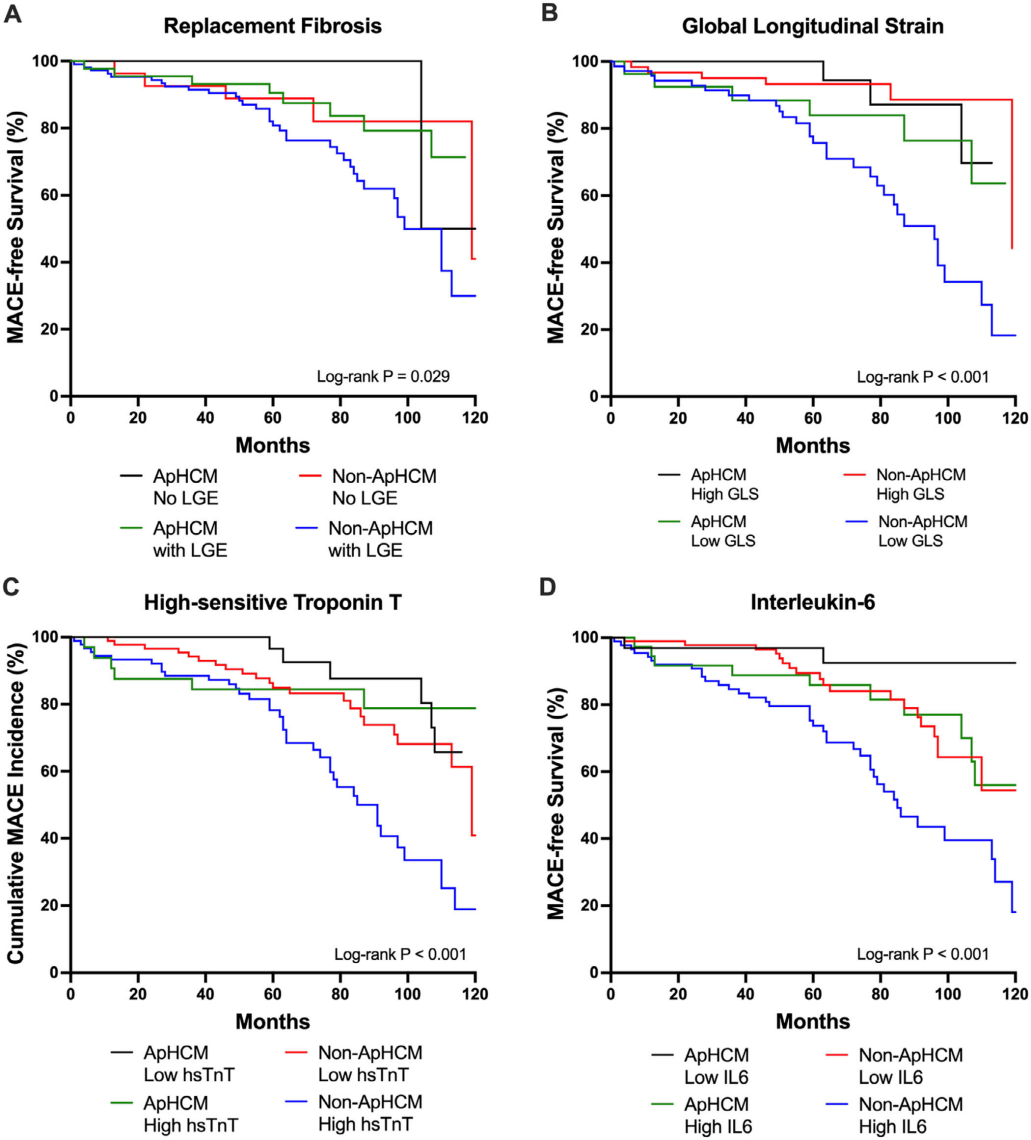
Major adverse cardiac event‐free survival in hypertrophic cardiomyopathy patients. Survival curves for major adverse cardiac events among apical hypertrophic cardiomyopathy and nonapical hypertrophic cardiomyopathy, with subgroup risk‐stratification by (A) presence of myocardial fibrosis, (B) global longitudinal strain below and above study mean, (C) high‐sensitive troponin T below and above median levels, and (D) interleukin‐6 below and above median levels.

These findings underscore the additive prognostic value of integrating IL‐6 levels with morphological classification in HCM. Non‐ApHCM patients with elevated IL‐6 levels exhibited the highest myocardial fibrosis burden and the worst clinical outcomes, likely reflecting a complex interplay between genetic predisposition and chronic inflammation. Notably, IL‐6 was elevated in many Non‐ApHCM patients without pathogenic sarcomere mutations, suggesting that chronic inflammation may represent a parallel risk pathway independent of genetic predisposition; however, larger cohorts are needed to test for an interaction between genotype and IL‐6.

Although CRP has been traditionally used as a marker of systemic inflammation in cardiovascular diseases, IL‐6 is more directly implicated in myocardial inflammation pathways relevant to HCM progression. Experimental studies further corroborate the mechanistic role of IL‐6 in driving pathological cardiac hypertrophy and fibrosis [5]. IL‐6 promotes fibroblast activation, enhances collagen deposition, and triggers maladaptive signaling cascades within cardiomyocytes. The absence of strong direct correlations between IL‐6 levels and structural imaging parameters in this cohort highlights the possibility that IL‐6 may precede visible myocardial damage, thus serving as an early warning signal for disease progression.

The integration of IL‐6 into existing HCM risk stratification models could enable more precise risk prediction and foster personalized management strategies. Targeted anti‐inflammatory therapies aimed at modulating IL‐6 signaling pathways may represent a promising avenue for therapeutic intervention in high‐risk HCM subsets.

This study's observational design precludes establishing causality between elevated IL‐6 levels, genetic mutations, and myocardial fibrosis. Although ROC‐derived cutoffs are clinically appealing as they maximize sensitivity and specificity, in our dataset, the ROC‐based threshold was nearly identical to the cohort median. This concordance supports the use of median as a robust and interpretable cutoff, while avoiding the potential overfitting of sample‐specific ROC thresholds. External validation in independent cohorts will be important to confirm the generalizability of these findings. Additionally, the relatively small number of ApHCM patients with relatively few events limits subgroup analyses. Larger, multicenter studies with longer follow‐up periods and wider cytokine panel are warranted to validate and extend these observations.

In conclusion, this is the first prospective Asian cohort study integrating IL‐6 with CMR phenotyping and HCM morphological subtypes, demonstrating that systemic inflammation provides independent prognostic value beyond genetics and imaging. Combining IL‐6 with CMR‐derived structural assessments and genotype information enhances risk stratification in Asian HCM patients and reveals a novel inflammatory risk pathway. These findings support the incorporation of inflammatory biomarkers into clinical management algorithms, potentially guiding the development of novel therapeutic strategies targeting inflammation in HCM.

## Author Contributions

T.T.L. conceived the study, oversaw data interpretation, and drafted the manuscript. S.L., C.Y., J.A.B., and Y.H. contributed to data collection, analysis, and visualization. S.K.P. and T.‐C.A. provided biomarker expertise. S.A.C. and C.W.‐L.C. supervised the study, secured funding and critically revised the manuscript, serving as joint senior authors. All authors have read and approved the final manuscript.

## Funding

The study was supported by the Ministry of Health and National Medical Research Council (NMRC/CG1/003/2021‐NHCS). The funder provided financial support but had no role in the study design, data collection, data analysis, interpretation of results, manuscript preparation, or decision to submit for publication.

## Ethics Statement

The study (ClinicalTrials.gov NCT02804269) was conducted in accordance with the Local Tissue Acts and the Declaration of Helsinki and approved by the SingHealth Centralized Institutional Review Board (CIRB 2019/2241). Written informed consent was obtained from all participants.

## Conflicts of Interest

The authors have nothing to report.

## Supporting information




**Supporting File S1**: mco270463‐sup‐0001‐SuppMat.docx

## Data Availability

The data that support the findings of this study are available from NHCS Biobank. Restrictions apply to the availability of these data, which were used under license for this study. Data are available with the permission of NHCS Biobank Committee at the National Heart Centre Singapore.
