# Supplementary material for: Circulating Interleukin‐6 Predicts Adverse Outcomes in Asians with Hypertrophic Cardiomyopathy
Source: MedComm (2020). 2025 Dec 8;6(12):e70463. doi: 10.1002/mco2.70463 (PMC12686114; doi:10.1002/mco2.70463)
Supplement: Supplementary file 1 — Supporting File S1: mco270463‐sup‐0001‐SuppMat.docx [file MCO2-6-e70463-s001.docx]

Supplementary Materials for

**Circulating Interleukin-6 Predicts Adverse Outcomes in Asians with Hypertrophic Cardiomyopathy**

Thu-Thao Le^1*^, Shiqi Lim^1^, Chengxi Yang^1^, Jennifer Ann Bryant^1^, Yiying Han^1^, Soon Kieng Phua^2^, Tar-Choon Aw^2^, Stuart Alexander Cook^1#^, Calvin Woon-Loong Chin^1#^

*Corresponding author: gmsltt@nus.edu.sg

**The file includes:**

Methods

References

**METHODS**

**Sequencing and variant classification**

All patients underwent targeted sequencing of HCM-associated genes using the Illumina TruSight Cardio panel, following established laboratory protocols [1]. Briefly, libraries were indexed, pooled, and sequenced (MiSeq, MiniSeq, NextSeq 500; 150 bp paired-end reads). Patients were categorised under four genetic strata. Understudied population including Singaporean HCM cases had previously found to have a greater excess of variant of unknown significance compared to the White population. Patients were classified as genotype-positive (G+) if carrying a rare protein-altering variant (allele frequency <0.00004) or recurrent local founder variants (TNNT2:p.Arg286His, TNNI3:p.Arg79Cys). G+ cases were subcategorized into thick filament (MYBPC3, MYH7, MYL2, MYL3), thin filament (ACTC1, TNNC1, TNNI3, TNNT2, TPM1), and other/“genocopy” genes (CSRP3, FHL1, GLA, PLN, PRKAG2, LAMP2). All other cases were considered genotype-negative (G−).

**Variant curation pipeline**

Reads were processed using GATK v3.8.1 HaplotypeCaller and mapped to GRCh37/hg19. Variants were annotated with Ensembl VEP v102, dbNSFP v4.1a, gnomAD v2.1.1, ClinVar (20210213), and LOFTEE. Pathogenicity was assigned according to ACMG/AMP guidelines.

**Serum biomarkers**

Blood samples were stored at –80°C. Serum NT-proBNP, hsTnT, and IL-6 were measured by electrochemiluminescence immunoassay; CRP by particle-enhanced immunoturbidimetry (Cobas C701 analyzer, Roche Diagnostics). The manufacturer-reported lower limit of detection was 5 pg/mL for NT-proBNP, 3 pg/mL for hsTnT, 0.3 mg/L for CRP, and 1.5 pg/mL for IL-6. Values below detection were imputed as half the limit.

**Cardiovascular magnetic resonance imaging**

A subset (n=196) underwent CMR on 1.5T Siemens Aera or 3T Philips Ingenia scanners. Cine images were acquired in long- and short-axis planes. Late gadolinium enhancement (LGE), native and post-contrast T1 mapping (MOLLI sequence) were performed with 0.1 mmol/kg gadobutrol.

CMR images were analyzed at the NHCS Core Laboratory using CVI42 by blinded readers. LV volumes, mass, maximal wall thickness (WTmax), and remodeling index (RI) were measured using standard protocols [2,3]. Myocardial strains were derived from cine images. Fibrosis was quantified by LGE, extracellular volume (ECV), and interstitial fibrosis volume from T1 mapping.

**Outcome definitions**

The primary endpoint was major adverse cardiac events (MACE: composite of ventricular arrhythmia, heart failure, atrial fibrillation, stroke, or all-cause mortality). Events were adjudicated by an independent cardiologist. Event rates were expressed per 100 patient-years.

**Statistical analysis**

Sample size of 255 patients was determined based on the expected event rate of MACE of approximately 20% over 5 years [4]. Continuous variables were expressed as mean±SD or median (IQR), categorical as n (%). Group comparisons used Student’s t test or ANOVA (parametric), Mann-Whitney U or Kruskal-Wallis (non-parametric), and χ² test for categorical data. Associations between predictors and CMR measures were assessed by linear regression adjusted for age, sex, comorbidities, and genotype.

Event-free survival was analyzed with Kaplan-Meier curves and log-rank test. Univariable and multivariable Cox proportional hazards models evaluated predictors of MACE. All models were adjusted for age and sex (in addition to HCM subtype and other significant covariates) to account for potential confounding of these factors. Proportional hazards assumption was checked using log-minus-log plots. ROC analysis was performed to identify optimal IL-6 cutoff; Youden’s index-based threshold was compared against the cohort median. Missing data were minimal and addressed using pairwise deletion in regression analyses. Statistical analyses were performed using SPSS v28 (IBM) and GraphPad Prism v10.2; p<0.05 was considered significant.

**REFERENCES**

1. Pua CJ, Bhalshankar J, Miao K, et al. Development of a Comprehensive Sequencing Assay for Inherited Cardiac Condition Genes. J Cardiovasc Transl Res. Feb 2016;9(1):3-11. doi:10.1007/s12265-016-9673-5

2. Le TT, Tan RS, De Deyn M, et al. Cardiovascular magnetic resonance reference ranges for the heart and aorta in Chinese at 3T. J Cardiovasc Magn Reson. 2016;18:21. doi:10.1186/s12968-016-0236-3

3. Le TT, Huang B, Pua CJ, et al. Lowering the Recommended Maximal Wall Thickness Threshold Improves Diagnostic Sensitivity in Asians With Hypertrophic Cardiomyopathy. JACC Asia. Sep 2021;1(2):218-226. doi:10.1016/j.jacasi.2021.07.001

4. Chen QF, Zou J, Katsouras CS, et al. Clinical Characteristics and Outcomes in Patients With Apical and Nonapical Hypertrophic Cardiomyopathy. Journal of the American Heart Association. 2024:e036663. doi:doi:10.1161/JAHA.124.036663
